# Olfactory Sensory Neurons Control Dendritic Complexity of Mitral Cells via Notch Signaling

**DOI:** 10.1371/journal.pgen.1006514

**Published:** 2016-12-27

**Authors:** Yuko Muroyama, Atsushi Baba, Motoo Kitagawa, Tetsuichiro Saito

**Affiliations:** 1 Department of Developmental Biology, Graduate School of Medicine, Chiba University, Chiba, Japan; 2 Department of Molecular and Tumor Pathology, Graduate School of Medicine, Chiba University, Chiba, Japan; New York University, UNITED STATES

## Abstract

Mitral cells (MCs) of the mammalian olfactory bulb have a single primary dendrite extending into a single glomerulus, where they receive odor information from olfactory sensory neurons (OSNs). Molecular mechanisms for controlling dendritic arbors of MCs, which dynamically change during development, are largely unknown. Here we found that MCs displayed more complex dendritic morphologies in mouse mutants of *Maml1*, a crucial gene in Notch signaling. Similar phenotypes were observed by conditionally misexpressing a dominant negative form of *MAML1* (*dnMAML1*) in MCs after their migration. Conversely, conditional misexpression of a constitutively active form of *Notch* reduced their dendritic complexity. Furthermore, the intracellular domain of Notch1 (NICD1) was localized to nuclei of MCs. These findings suggest that Notch signaling at embryonic stages is involved in the dendritic complexity of MCs. After the embryonic misexpression of *dnMAML1*, many MCs aberrantly extended dendrites to more than one glomerulus at postnatal stages, suggesting that Notch signaling is essential for proper formation of olfactory circuits. Moreover, dendrites in cultured MCs were shortened by Jag1-expressing cells. Finally, blocking the activity of Notch ligands in OSNs led to an increase in dendritic complexity as well as a decrease in NICD1 signals in MCs. These results demonstrate that the dendritic complexity of MCs is controlled by their presynaptic partners, OSNs.

## Author Summary

Olfactory circuits are critical for the survival of many animals. Odor information is transmitted from olfactory sensory neurons (OSNs) to relay neurons, the morphology of which is crucial for processing of the information and similar among species. The major relay neurons, mitral cells (MCs) in mammals and projection neurons in flies, have a single primary dendrite at the mature stage. Molecular mechanisms to control the formation of the dendrite are largely unknown. MCs dynamically change their dendrites during development. In this study, we show that the dendritic morphologies of MCs are controlled by Notch signaling, many factors of which are well conserved among species. Moreover, we have found that Notch signaling in MCs is activated by OSNs, and that Notch operates in the relay neurons in the mouse olfactory system, in contrast to the fly system, where Notch functions in OSNs. Therefore, our study has revealed a novel step for shaping the dendritic morphologies of MCs.

## Introduction

Dendritic morphologies of neurons, which are crucial for receiving information from presynaptic neurons and characteristic of neuronal types, are controlled by various extracellular and intracellular factors [1–3]. The dendrites of *Drosophila* dendritic arborization neurons, which are a well-characterized model system, have been recently shown to be regulated by epidermis-secreted Sema-2b protein [4] and transcriptional programs [5]. Dendrites in neurons of the cerebral cortex also involve transcriptional regulators, such as Neurog2 [6] and Notch [7,8]. However, it is difficult to determine precisely when, where and how those factors control dendritic morphologies in the cerebral cortex, because they are functional at multiple stages, and there are numerous neuronal types in the cerebral cortex [9]. Neurog2 controls neuronal differentiation and migration as well as the specification of neuronal types [6]. Notch has been suggested to be involved in the migration and morphogenesis of differentiated neurons as well as the maintenance of neural progenitor and stem cells [10]. Neuronal types are changed by transient activation of Notch in neural progenitor cells [11].

In contrast to the cerebral cortex, the olfactory bulb (OB) has a simpler structure and only two types of projection neurons, mitral cells (MCs) and tufted cells. MCs have been well studied. In the developing mouse embryo, most MCs are generated in the ventricular zone from embryonic day (E) 10.5 to E13.5, migrate to the MC layer of the OB and extend multiple dendrites [12–14]. MCs increase the number and length of their dendrites during embryonic stages [13]. At postnatal stages, MCs come to display the mature morphology, in which a single primary dendrite extends into a single glomerulus [13,15,16]. However, molecular mechanisms to control their dendritic morphologies are poorly understood, although the projection of olfactory sensory neuron (OSN) axons to MCs has been intensively investigated [17,18]. Knockout mice of *Cnga2*, which is expressed in OSNs, has been reported to show slowed dendritic pruning of MCs as wells as reduced body size [15].

*Maml1*^−/−^ mice die soon after birth [19,20] and have defects in the development of lymphocytes [20,21] and muscles [19]. The Maml1 protein cooperates with the DNA-binding protein Rbpjκ and the intracellular domain of Notch1 (NICD1), which is generated by the cleavage of Notch1 upon binding to ligands, such as Jag1, to activate transcription of target genes [22]. Clear phenotypes have not been reported in the nervous system of *Maml1* mutants.

Here, we found that MCs exhibited increased dendritic complexity in *Maml1* mutant mice in a gene dose-dependent manner at E18.5 but not E15.5. At postnatal day (P) 9, at which stage most MCs had the mature morphology in *Maml1*^+/+^ mice, many MCs extended dendrites into more than one glomerulus in *Maml1*^+/−^ mice, and some *Maml1*^+/−^ mice were defective in homing behavior. Using *in vivo* electroporation and the tetracycline (Tet)-controlled gene expression system, we modulated the activity of Notch signaling in MCs after their birth and migration in the developing mouse embryo. Whereas misexpression of a dominant negative form of *MAML1* (*dnMAML1*) phenocopied *Maml1* mutants, a constitutively active form of *Notch* (*caNotch*) reduced dendritic complexity in MCs. Moreover, the number and length of dendrites in cultured MCs were decreased when cocultured with Jag1-overexpressing cells but not with control cells. These findings suggest that the dendritic complexity of MCs is controlled by Notch signaling at embryonic stages. Furthermore, misexpression of *dnMAML1* at embryonic but not postnatal stages led to many aberrant MCs, which extended dendrites to more than one glomerulus at P9, suggesting that embryonic Notch signaling is essential for proper formation of olfactory circuits. Furthermore, NICD1 was observed in nuclei of MCs in embryos. Misexpression in OSNs of a dominant negative form of *Mib1* (*dnMib1*), which inhibits the activity of Notch ligands, led to increased dendritic complexity as well as a decrease in NICD1 in nuclei in MCs.

## Results

### MCs exhibited increased dendritic complexity in *Maml1* mutants

Because *Maml1*^−/−^ mice die soon after birth [19,20], we investigated *Maml1*-deficient embryos at E18.5 (S1 Fig). The sizes of the OB and cerebral cortex were indistinguishable among *Maml1*^+/+^, *Maml1*^+/−^ and *Maml1*^−/−^ embryos. No gross abnormalities in the lamination of the OB were detected in *Maml1*-deficient embryos.

**Fig 1:**
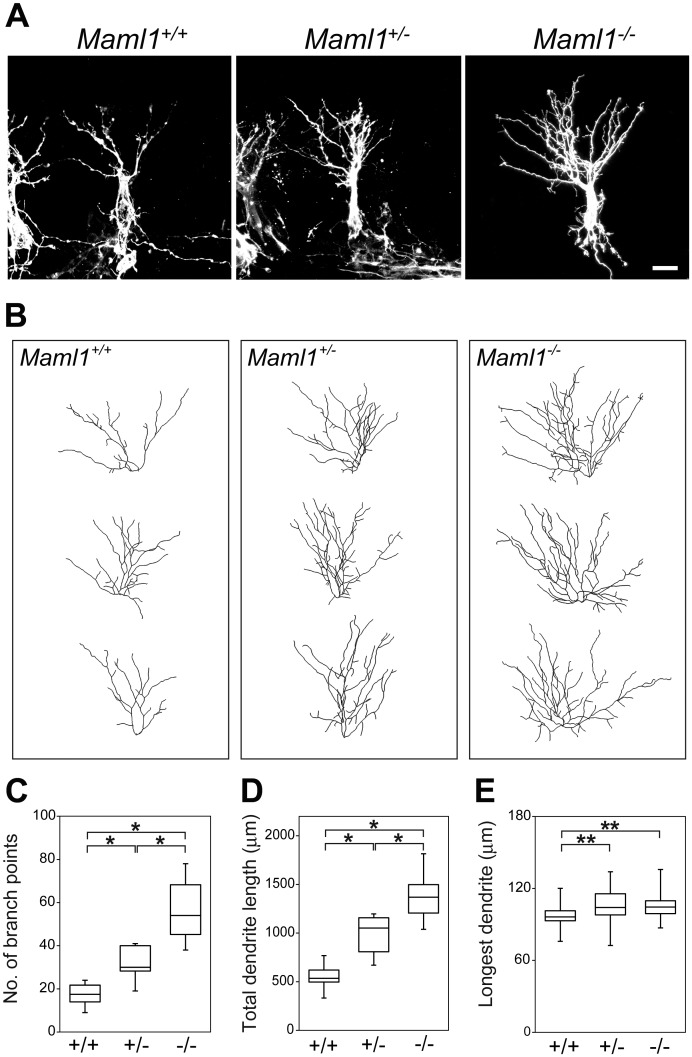
Dendritic complexity of MCs at E18.5. (**A, B**) Representative images (**A**) and dendritic tracing (**B**) of DiI-labeled MCs in *Maml1*^+/+^, *Maml1*^+/−^ and *Maml1*^−/−^ mice at E18.5. Scale bar: 20 μm. (**C–E**) Number of branch points (**C**), total dendrite length (**D**) and length of the longest dendrite (**E**) of MCs: *Maml1*^+/+^, *n* = 22 from 5 mice; *Maml1*^+/−^, *n* = 18 from 5 mice; *Maml1*^−/−^, *n* = 18 from 4 mice. MCs that were in the dorsomedial OB and located at 300 to 700 μm from the rostral tip of the OB were used for the analyses. The phenotypes were indistinguishable among embryos of the same genotype. Box plots indicate the median, 25th/75th percentiles (box) and the data range. *P < 0.005, **P < 0.05.

We examined MCs by labeling them with DiI injection into the lateral olfactory tract (LOT), which contains axons of MCs. Strikingly, MCs exhibited more complex dendritic morphologies in *Maml1*^−/−^ and *Maml1*^+/−^ than *Maml1*^+/+^ embryos (Fig 1A). To determine dendritic complexity, 3D structures of dendrites were reconstructed using the FilamentTracer software (Fig 1B), and the number of branch points, total dendrite length and length of the longest dendrite were quantified (Fig 1C–1E). They were significantly increased in *Maml1*^+/−^ and *Maml1*^−/−^ embryos. *Maml1*^+/−^ embryos demonstrated an intermediate phenotype between *Maml1*^+/+^ and *Maml1*^−/−^ embryos, indicating that the dendritic complexity of MCs is affected by the dosage of the *Maml1* gene.

**Fig 2:**
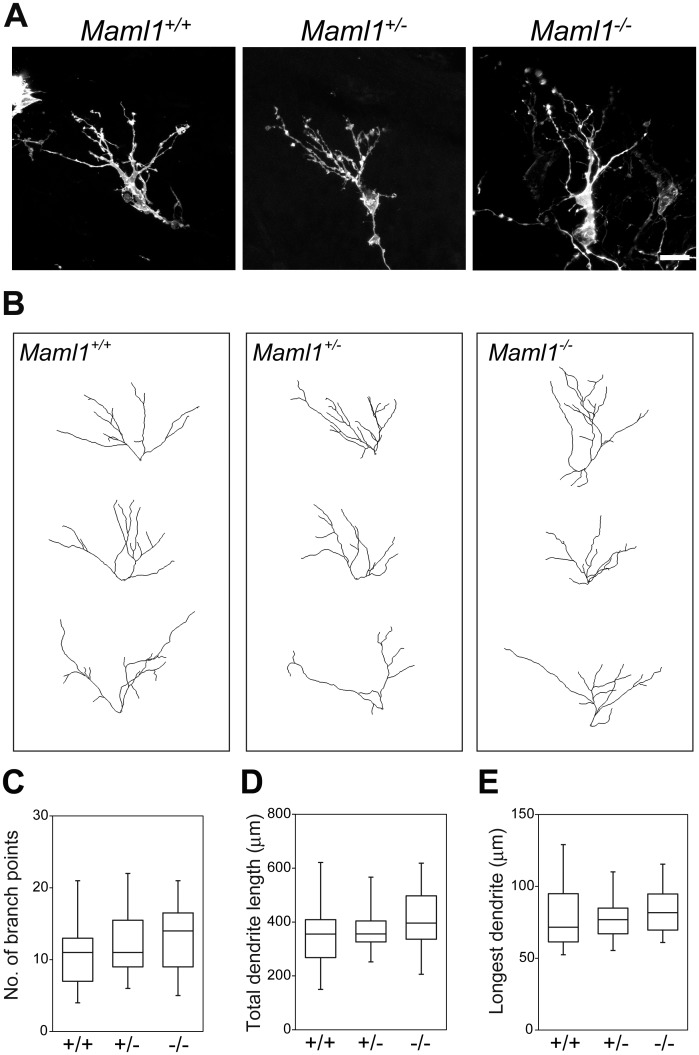
Dendritic complexity was not affected in MCs of *Maml1* mutants at E15.5. (**A, B**) Representative images (**A**) and dendritic tracing (**B**) of MCs at E15.5. Scale bar: 20 μm. (**C–E**) Number of branch points (**C**), total dendrite length (**D**) and length of the longest dendrite (**E**) of MCs: *Maml1*^+/+^, *n* = 25 from 6 mice; *Maml1*^+/−^, *n* = 23 from 5 mice; *Maml1*^−/−^, *n* = 22 from 5 mice. MCs that were in the dorsomedial OB and located at 300 to 600 μm from the rostral tip of the OB were analyzed as in Fig 1. There were no significant differences in the number of branch points, total dendrite length or length of the longest dendrite among the genotypes (P ranged from 0.181 to 0.728).

To learn which stage is critical for the mutant phenotypes, we examined embryos at E15.5, at which stage many MCs have already finished radial migration to the MC layer and extended multiple dendrites [13]. No significant differences in the number of branch points, total dendrite length or length of the longest dendrite were detected in *Maml1*-deficient embryos (Fig 2), suggesting that the E18.5 mutant phenotypes were caused after the migration and initial dendrite outgrowth of MCs.

### Conditional misexpression in MCs

To modulate gene expression in MCs at specific stages, we used *exo utero* electroporation [23,24] at E11.5, which was effective for transfection into MCs (S2 Fig), and the Tet-controlled gene expression system [25]. After cotransfection with CAG-rtTA, which carries the reverse Tet-controlled transcriptional activator downstream of the ubiquitous promoter CAG, a gene downstream of the Tet-responsive element (TRE) was specifically induced in the presence of doxycycline (Dox) (S3 Fig).

### *caNotch* and *dnMAML1* altered dendritic complexity in MCs

**Fig 3:**
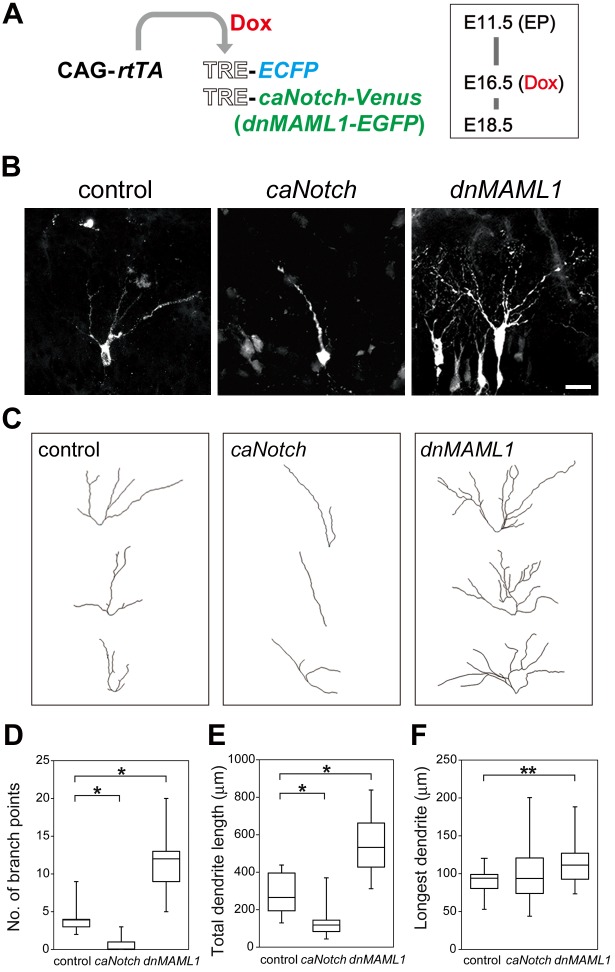
Conditional misexpression of *caNotch* or *dnMAML1* altered dendritic complexity in MCs. (**A**) Illustration of electroporation (EP) of CAG-*rtTA* and TRE-*ECFP* (control), and CAG-*rtTA*, TRE-*ECFP* and TRE-*caNotch*-*Venus* or TRE-*dnMAML1*-*EGFP* at E11.5, induction by Dox at E16.5 and analysis at E18.5. (**B, C**) Representative images (**B**) and dendritic reconstructions (**C**) of MCs, which misexpressed *ECFP* alone (control) and *ECFP* with *caNotch-Venus* (*caNotch*) or *dnMAML1-EGFP* (*dnMAML1*). MCs were visualized using an anti-GFP antibody that reacts with ECFP. Scale bar: 20 μm. (**D–F**) Anti-GFP antibody-stained dendrites were analyzed as in Fig 1: control, *n* = 21 from 5 mice; *caNotch*, *n* = 21 from 5 mice; *dnMAML1*, *n* = 21 from 6 mice. The phenotypes were indistinguishable among embryos transfected with the same genes. *P < 0.005, **P < 0.05.

To examine whether *Maml1* and the Notch pathway in MCs were crucial for their dendritic structures, we conditionally misexpressed *caNotch* [23] or *dnMAML1* [26] in MCs at E16.5 (Fig 3A), at which stage MCs had already settled in the MC layer [13]. MCs positive for Venus or enhanced green fluorescent protein (EGFP), which was coexpressed with *caNotch* or *dnMAML1*, were chosen for analyses. TRE-*enhanced cyan fluorescent protein* (*ECFP*) was cotransfected to label cells, because nuclear localization of dnMAML1-EGFP made it difficult to match dnMAML1-EGFP-positive cells with DiI-labeled dendrites. 3D structures of dendrites were reconstructed as above. The number of branch points and total dendrite length were smaller than those of DiI-labeled MCs in Fig 1, probably because DiI spreads in the plasma membrane and is more sensitive in detecting thin membranous protrusions than ECFP, which is a cytoplasmic protein. Compared to misexpression of *ECFP* alone as a control, *caNotch* drastically reduced the number of branch points and total dendrite length (Fig 3B–3E). Conversely, *dnMAML1* significantly increased them. The length of the longest dendrite was changed by misexpression of *dnMAML1* (Fig 3F). These findings indicate that the Notch pathway in MCs is responsible for their dendritic complexity after their migration.

### The number and length of dendrites were reduced by presentation of Jag1 *in vitro*

**Fig 4:**
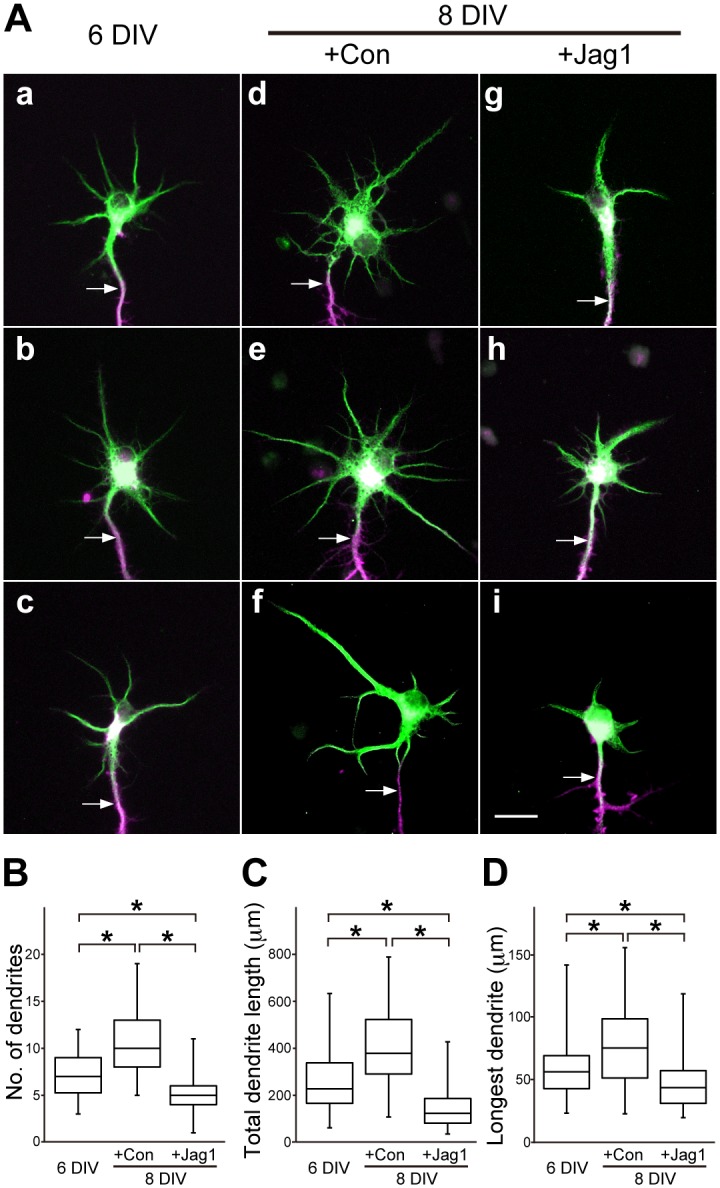
Decrease of the number and length of dendrites by Jag1-overexpressing cells. (**A**) Representative images of cultured Tbx21-positive OB neurons immunostained with MAP2 (green) and Tau-1 (magenta) at 6 DIV (**a–c**) and two days after coculture with the control cells (+Con) (**d–f**) or Jag1-overexprresing cells (+Jag1) (**g–i**). Arrows indicate axons. Scale bar: 20 μm. (**B–D**) Number of dendrites (**B**), total dendrite length (**C**) and length of the longest dendrite (**D**) of the neurons. Data are from four independent preparations: 6 DIV, *n* = 58; +Con, *n* = 57; +Jag1, *n* = 62. Similar effects were observed in all preparations. *P < 0.005.

To evaluate whether a Notch ligand was able to directly affect neurons, dissociated cells were cultured at low density from the OB at E15.5, at which stage all MCs and some tufted cells have been already generated [12–14]. The vast majority (95.28 ± 1.28%) of the cells (*n* = 3365) were neurons and mostly not in contact with other cells. Almost all (99.76 ± 0.40%) of the neurons were positive for Tbx21, a marker of MCs and tufted cells [27,28], and extended multiple MAP2-positive dendrites and a single Tau-1-positive axon at 6 days *in vitro* (DIV) (Fig 4Aa–4Ac). MCs and tufted cells are aligned in different layers of the OB but are indistinguishable *in vitro*. They were cocultured for two days with control Nalm-6 cells (Fig 4Ad–4Af) or Nalm-6 cells overexpressing Jag1 (Fig 4Ag–4Ai), which activate the Notch pathway *in vitro* [29], and Tbx21-positive neurons were analyzed using the FilamentTracer software. The number of dendrites, total dendrite length and length of the longest dendrite of the neurons treated with the Jag1-overexpressing cells were smaller than those treated with the control cells, suggesting that Jag1 directly affects neurons but not through other cell types, such as glia.

### Embryonic Notch signaling perturbation led to abnormal dendrites at postnatal stages

**Fig 5:**
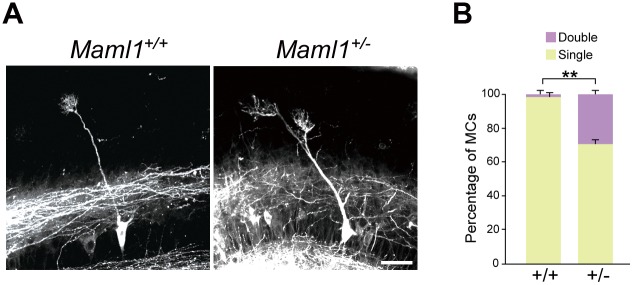
Abnormal dendrites of MCs in *Maml1*^+/−^ mice at P9. (**A**) Representative images of MCs at P9. Dendrites extending into two glomeruli branched in the superficial (**A**) and deep (see S7 Fig) external plexiform layers in *Maml1*^+/−^ mice. Scale bar: 50 μm. (**B**) Proportions of MCs with a primary dendrite extending into single or double glomeruli. MCs were labeled with DiI as in Fig 1. MCs that were in the dorsomedial OB and located at 500 to 900 μm from the rostral tip of the OB were used for the analyses. The phenotypes were indistinguishable among embryos of the same genotype. Error bars indicate SD. **P < 0.05.

At P9, most MCs display the mature morphology in wild type mice. Although no gross abnormalities were detected in the OB of *Maml1*^+/−^ mice at this stage (S4 Fig), a significant number (29.2 ± 2.6%) of MCs (*n* = 93 from 5 mice) had abnormal dendrites targeting more than one glomerulus in *Maml1*^+/−^ mice, whereas only a small number (1.1 ± 2.5%) of MCs (*n* = 73 from 5 mice) did in *Maml1*^+/+^ mice (Fig 5).

**Fig 6:**
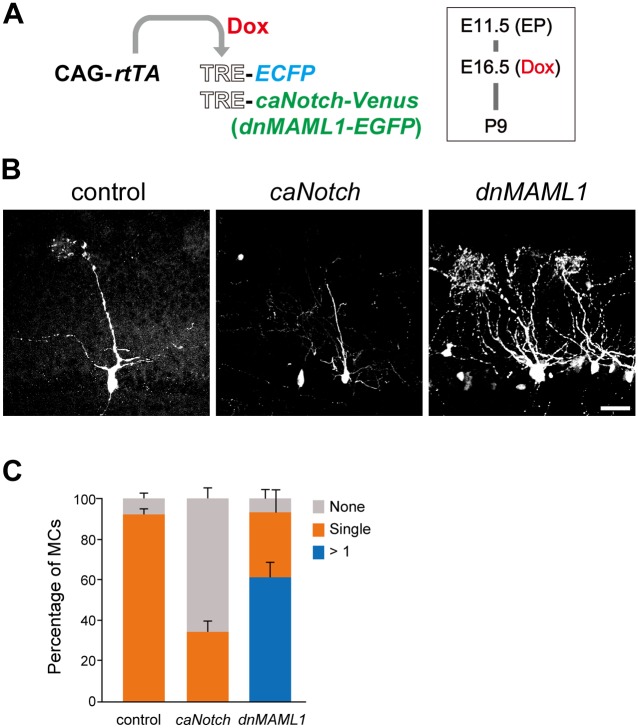
Embryonic perturbation of Notch signaling led to aberrant MCs at P9. (**A**) Illustration of EP at E11.5 and induction by Dox at E16.5 as described in Fig 3, and analysis at P9. (**B**) Representative images of MCs, in which *ECFP* alone (control) and *ECFP* with *caNotch-Venus* (*caNotch*) or *dnMAML1-EGFP* (*dnMAML1*) were misexpressed. MCs were analyzed as in Fig 3. The phenotypes were indistinguishable among embryos transfected with the same genes. Scale bar: 50 μm. (**C**) Proportions of MCs extending a primary dendrite into a single glomerulus (orange bar). Error bars indicate SD. The percentages of MCs extending dendrites into more than one glomerulus (blue bar) and MCs with a truncated primary dendrite (grey bar) were significantly increased by *dnMAML1* and *caNotch*, respectively: control and *caNotch*, P < 0.05; control and *dnMAML1*, P < 0.05.

To examine whether the aberrant morphology of MCs involved embryonic Notch signaling in MCs, MCs were analyzed at P9 after misexpression of *caNotch* or *dnMAML1* at E16.5 (Fig 6). Whereas almost all (92.2 ± 2.8%) of MCs (*n* = 56 from 4 mice) normally extended a single primary dendrite into a single glomerulus after misexpression of *ECFP* alone as a control, many MCs (61.4 ± 7.3%, *n* = 72 from 5 mice) extended multiple dendrites into more than one glomerulus after *dnMAML1* misexpression. On the other hand, many MCs (65.3 ± 5.3%, *n* = 69 from 4 mice) had a single primary dendrite that did not reach any glomeruli after *caNotch* misexpression, suggesting that excess Notch signaling is deleterious to MCs. These findings suggest that proper strength of embryonic Notch signaling is necessary for establishing the mature morphology of MCs.

**Fig 7:**
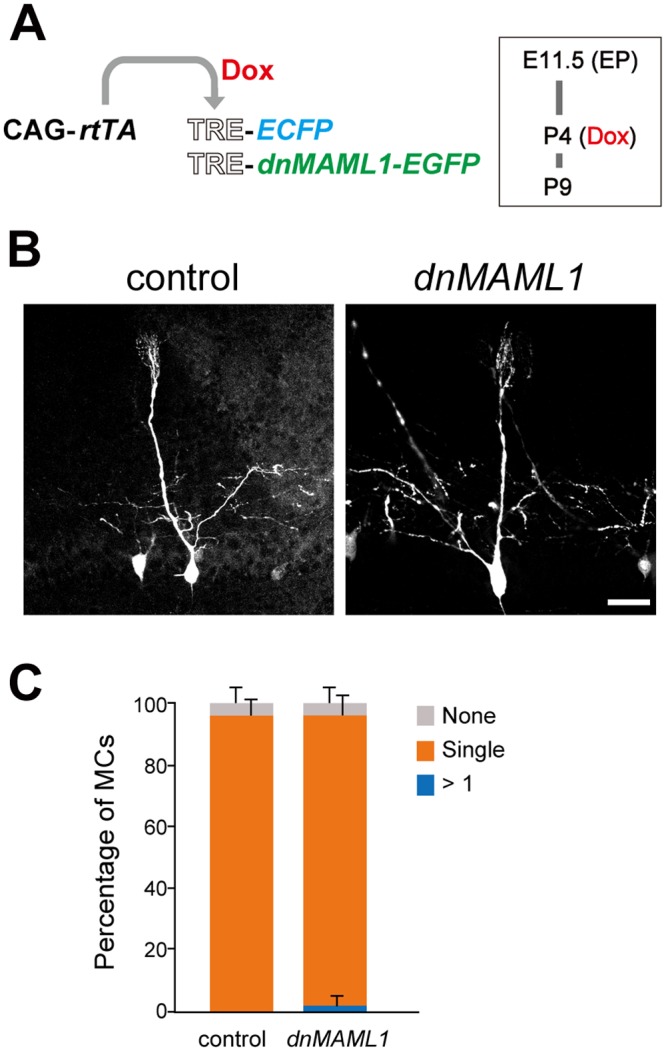
Notch signaling perturbation at P4 did not affect MCs. (**A**) Illustration of EP at E11.5 as described in Fig 3, induction by Dox at P4 and analysis at P9. (**B**) Representative images of MCs, in which *ECFP* alone (control) and *ECFP* with *dnMAML1-EGFP* (*dnMAML1*) were misexpressed. MCs were analyzed as in Fig 3. Scale bar: 50 μm. (**C**) Proportions of MCs extending a primary dendrite into a single glomerulus (orange bar). Error bars indicate SD. No significant changes in the percentages of MCs extending dendrites into more than one glomerulus (blue bar) or MCs with a truncated primary dendrite (grey bar) were detected between control and *dnMAML1* (P ranged from 0.240 to 0.893).

Next we examined whether postnatal perturbation of Notch signaling affected MCs, by inducing *dnMAML1* at P4, at which stage many MCs still extend multiple dendrites [13,15,30]. MCs were not changed by the postnatal misexpression (Fig 7). Nearly all (94.2 ± 6.3%) of MCs (*n* = 62 from 5 mice) had a single primary dendrite targeting into a single glomerulus after *dnMAML1* misexpression, equivalently to *ECFP* misexpression as a control (96.0 ± 5.3%, *n* = 51 from 4 mice). These findings suggest that Notch signaling is involved in early but not later stages of dendritic development in MCs.

Moreover, strong NICD1 signals were observed in MCs at E18.5 but not E15.5 or P4 (S5 Fig), suggesting that the canonical Notch pathway is active in MCs at E18.5.

### OSNs activated the canonical Notch pathway in MCs

**Fig 8:**
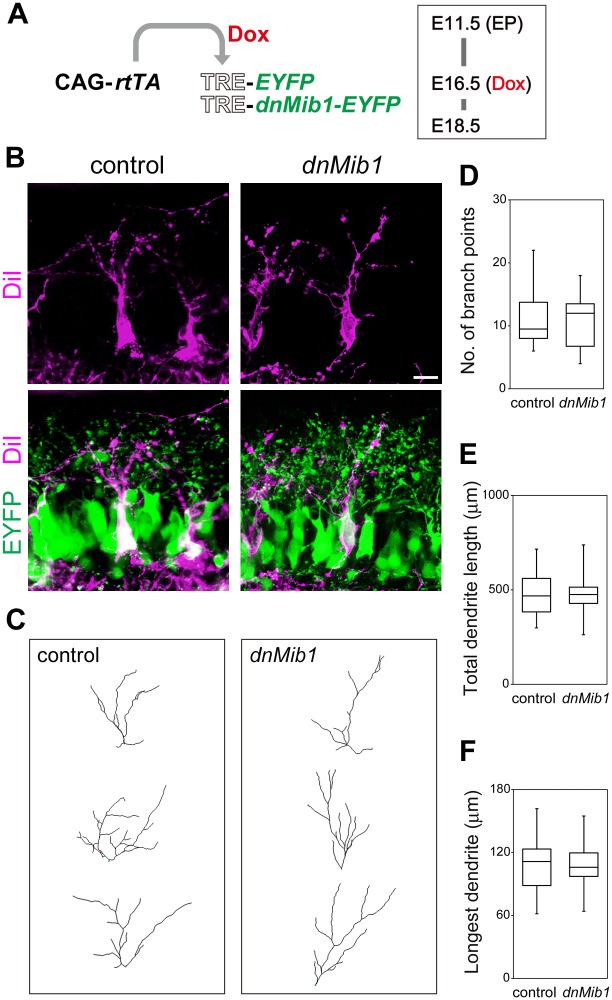
Dendritic complexity of MCs that misexpressed *dnMib1* was not altered. (**A**) Illustration of EP of CAG-*rtTA* and TRE-*EYFP* without (control) or with TRE-*dnMib1*-*EYFP* (*dnMib1*) at E11.5, induction by Dox at E16.5 and analysis at E18.5. (**B–F**) Representative images (**B**), dendritic reconstructions (**C**) and dendrite analyses of MCs that were positive for EYFP (green) (**D–F**). MCs were labeled with DiI and analyzed as in Fig 1: control, *n* = 18 from 6 mice; *dnMib1*, *n* = 16 from 6 mice. The phenotypes were indistinguishable among embryos transfected with the same genes. Scale bar: 20 μm. There were no significant differences in the number of branch points, total dendrite length or length of the longest dendrite of MCs between control and *dnMib1* (P ranged from 0.905 to 0.972).

To determine which cells provided a signal to activate the Notch pathway in MCs, we conditionally misexpressed *dnMib1*, which inhibits the function of all the canonical Notch ligands, Delta and Jagged [31,32]. Because the Notch pathway is often activated between neighboring cells [10], we first misexpressed *dnMib1* in MCs, by cotransfecting TRE-*dnMib1*-*EYFP* (*enhanced yellow fluorescent protein*) with CAG-*rtTA* at E11.5 and inducing its expression at E16.5 (Fig 8A), as performed in Fig 3 and S3 Fig. DiI retrograde labeling was used to visualize MCs, and 3D dendrite structures were reconstructed as above. MCs that misexpressed *dnMib1* normally extended dendrites at E18.5 (Fig 8B and 8C). The number of branch points, total dendrite length and length of the longest dendrite of MCs that misexpressed *dnMib1* were not significantly different from those which misexpressed *EYFP* alone as a control (Fig 8D–8F), suggesting that Notch is not activated by ligands that are expressed in the same MCs.

**Fig 9:**
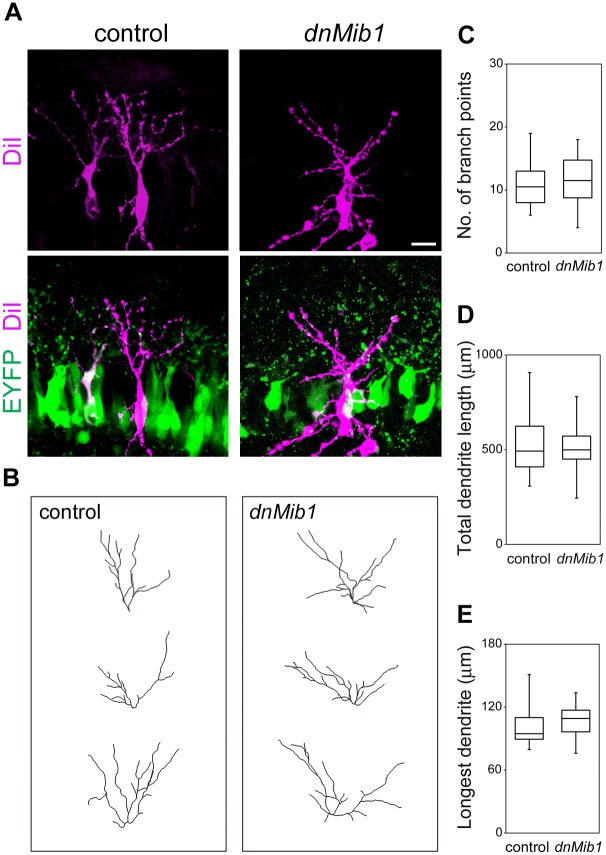
Dendritic complexity of MCs was not affected by neighboring MCs that misexpressed *dnMib1*. Representative images (**A**), dendritic reconstructions (**B**) and dendrite analyses of MCs that were not positive for EYFP (green) (**C–E**). MCs were electroporated and analyzed as in Fig 8: control, *n* = 20 from 5 mice; *dnMib1*, *n* = 20 from 5 mice. The phenotypes were indistinguishable among embryos transfected with the same genes. Scale bar: 20 μm. There were no significant differences in the number of branch points, total dendrite length or length of the longest dendrite of MCs between control and *dnMib1* (P ranged from 0.301 to 0.957).

MCs that did not misexpress *dnMib1* and were adjacent to those which misexpressed *dnMib1* were also not affected (Fig 9A and 9B). Their number of branch points, total dendrite length and length of the longest dendrite were not significantly changed (Fig 9C–9E). These findings suggest that Notch ligands that activate the Notch pathway in MCs are provided by cells other than MCs.

**Fig 10:**
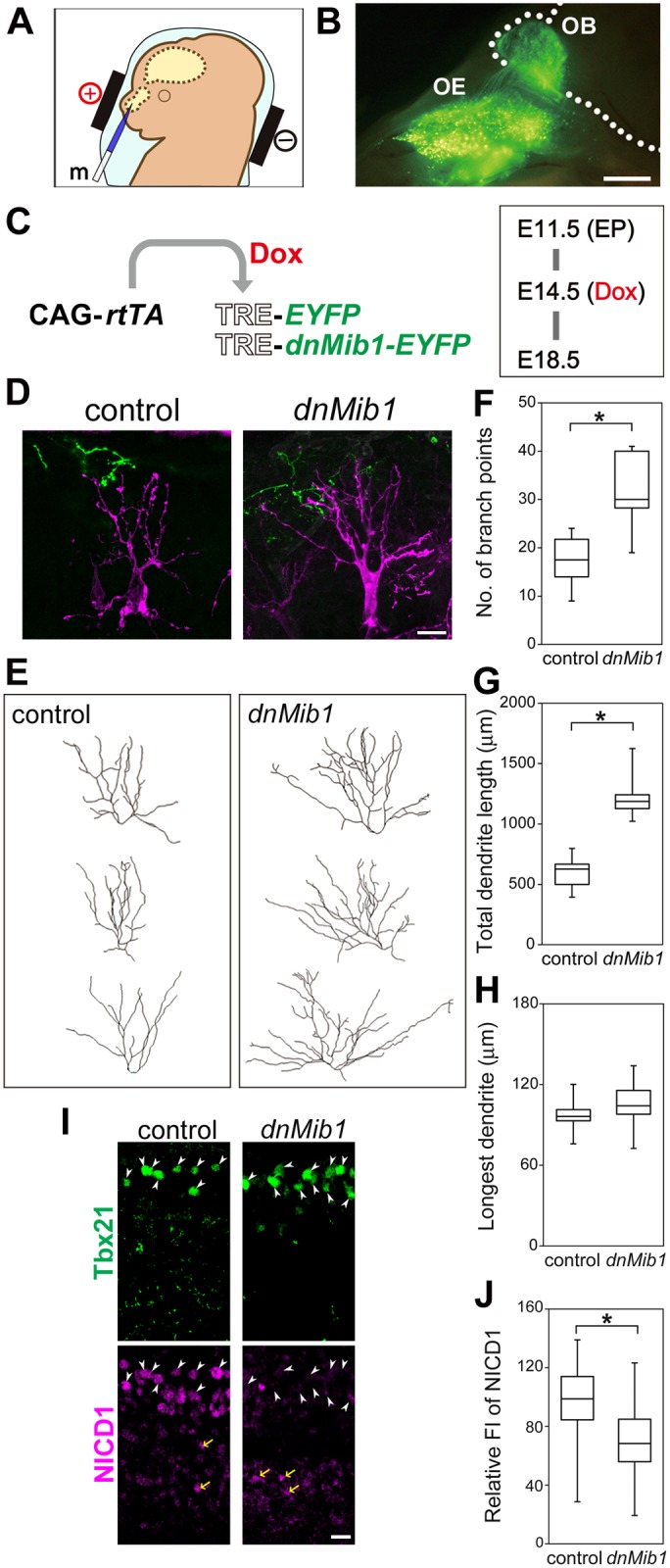
Misexpression of *dnMib1* in OSNs affected dendritic complexity of MCs. (**A**) Schematic illustration of transfection into the OE. (**B**) EYFP expression in the OE and OSN axons reaching the OB, 7 days after electroporation of CAG-*EYFP*. Numerous OSNs in the nasal cavity and their axons but none of MCs were labeled with EYFP by electroporation at E11.5. (**C**) Illustration of EP of CAG-*rtTA* and TRE-*EYFP* without (control) or with TRE-*dnMib1*-*EYFP* (*dnMib1*) at E11.5, induction by Dox at E14.5 and analysis at E18.5. (**D–H**) Representative images (**D**), dendritic reconstructions (**E**) and dendrite analyses of MCs (magenta) that were adjacent to EYFP-positive OSN axons (green) (**F–H**). MCs were analyzed as in Fig 1: control, *n* = 18 from 5 mice; *dnMib1*, *n* = 18 from 6 mice. (**I**) Immunostaining of Tbx21 (green) and NICD1 (magenta). The white arrowheads and yellow arrows indicate Tbx21-positive nuclei in the MC layer and Tbx21-negative NICD1-positive nuclei below the MC layer, respectively. Similar staining patterns were reproducibly observed in all examined embryos (*n* = 3 mice). The ventricle is to the bottom. (J) Relative FI of NICD1 in Tbx21-positive nuclei after conditional misexpression of *EYFP* (control) and *dnMib1* in OSNs. Control (*n* = 98 cells from four OBs) and *dnMib1* (*n* = 81 cells from three OBs). Scale bars: 0.5 mm in **B**; 20 μm in **D** and **I**. *P < 0.005.

Many OSN axons reach the OB at E14.5 [13,33]. To examine whether OSNs activated Notch in MCs, the olfactory epithelium (OE) was electroporated at E11.5 (Fig 10A and 10B), at which stage many OSNs are generated [34], and *dnMib1* was induced at E14.5 (Fig 10C).

*dnMib1* misexpression in OSNs increased dendritic complexity in MCs, compared with *EYFP* alone as a control (Fig 10D and 10E). The number of branch points and total dendrite length were significantly increased (Fig 10F and 10G). These results suggest that OSNs provide a signal(s) that controls the dendritic complexity of MCs.

Furthermore, NICD1 signals of Tbx21-positive cells were significantly decreased by *dnMib1* misexpression in OSNs (Fig 10I and 10J). There were some Tbx21-negative but NICD1-positive cells in the MC layer (Fig 10I), probably reflecting that some MCs are negative for Tbx21 at this stage [28]. NICD1 signals of Tbx21-negative cells in the MC layer appeared to be also decreased. These findings suggest that OSNs directly activate the canonical Notch pathway in MCs.

### Homing behavior of *Maml1* mutants

**Fig 11:**
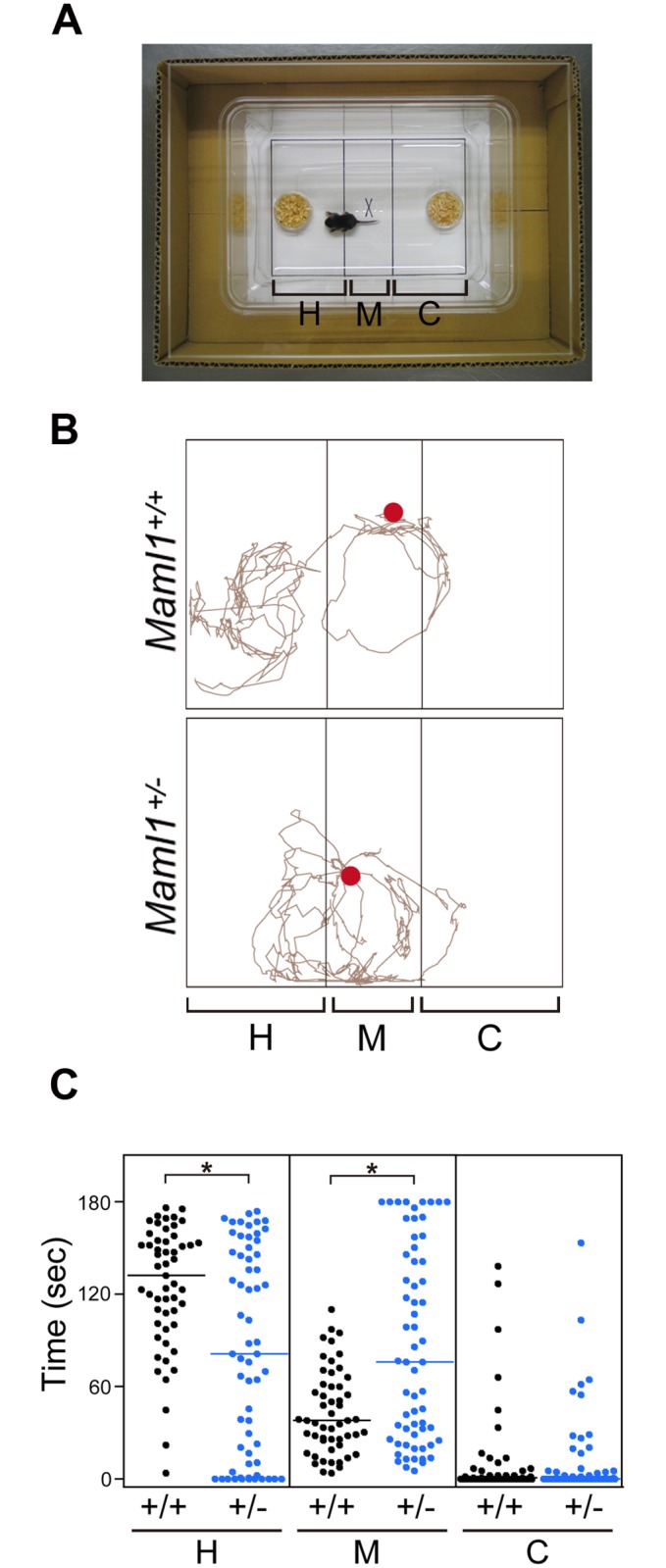
Homing behavior of *Maml1*^+/−^ mice. (**A**) Homing test cage divided into three areas. The H and C areas contained wood chips from the littermates’ home cage and unused chips, respectively. (**B**) Examples of pups’ movements in the cage. Red dots indicate pups’ positions at the start of video tracking. (**C**) Cumulative time spent in each area during the 3-min test. Each dot represents an individual *Maml1*^+/+^ (*n* = 53) or *Maml1*^+/−^ (*n* = 62) pup. Lines are median. *P < 0.005.

To learn whether *Maml1* mutants have defects in processing of olfactory information at P9, we examined homing behavior, in which mouse pups show preferences for nest-specific odors [35,36]. Most *Maml1*^+/+^ mice preferentially went to and stayed on the side containing wood chips from their home cage, after they were placed in the middle of a test cage in which unused chips were present on the opposite side (Fig 11). Many *Maml1*^+/−^ mice spent less time on the side containing their home cage chips, although their walking appeared to be normal. 5-cm walk was completed in 1.02 ± 0.12 s by *Maml1*^+/+^ mice (*n* = 191) and in 1.03 ± 0.17 s by *Maml1*^+/−^ mice (*n* = 209). These findings suggest that some *Maml1*^+/−^ mice have defects in the processing of nest-specific odors.

## Discussion

**Fig 12:**
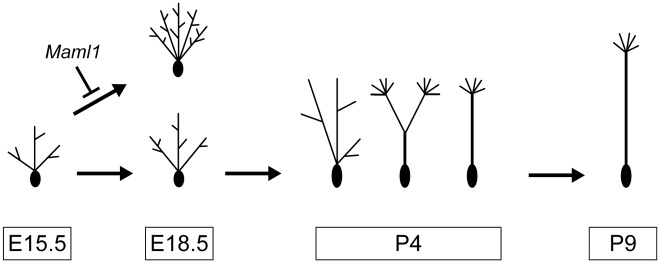
Schematic illustration of the development of MCs from multiple dendrites to a single primary dendrite.

MCs change their dendritic morphologies during development (Fig 12; [13,15,30]). MCs extend multiple dendrites at embryonic stages. At early postnatal stages, such as P4, some dendrites start to form glomerular tufts at their termini, and three types of MCs, which have multiple dendrites (embryo type), a primary dendrite with tufts in more than one glomerulus (transit type) and a single primary dendrite with a tuft only in a single glomerulus (mature type), are observed. The control of dendrite forms in embryonic MCs has been completely overlooked, because their dendrites continue to grow at embryonic stages. The effects of the Notch pathway on their dendritic morphologies has not been addressed, although Notch1 has been shown to be expressed in MCs, periglomerular cells and granule cells in the adult OB, and has been implicated in olfactory information processing in adult mice by using *CamKII*-*Cre*-mediated *Notch1* knockout mice [37]. Here, we report for the first time that the dendritic complexity of embryonic MCs is controlled by the canonical Notch pathway, which is activated by their presynaptic partners, OSNs, and that Notch signaling is crucial for the formation of mammalian olfactory circuits.

*Drosophila* olfactory circuits have been shown to involve Notch, which controls the diversification of OSNs [38] and volume of glomeruli [39]. The *Drosophila* Notch pathway, however, operates in OSNs [39] and their precursors [38]. Thus, the cells in which Notch works in *Drosophila* are on the presynaptic side, in clear contrast to the postsynaptic side in the mouse that we report here.

In our cell culture assay, the number of dendrites, total dendrite length and length of the longest dendrite of the Tbx21-positive neurons were significantly decreased by the Jag1-overexpressing cells in comparison with 6 DIV (Fig 4B–4D), suggesting that Notch signaling might be involved not only in dendritic growth inhibition but also pruning. All the neurons appeared to respond similarly to the Jag1-overexpressing cells, suggesting that Notch signaling may also control tufted cells, which have a single primary dendrite extending into a single glomerulus [40].

It has been shown that dendritic morphologies of MCs are normal until P3 but their dendritic pruning from P4 to P6 is slowed in *Cnga2* knockout mice, although the phenotype may be linked with a secondary effect by reduced body size [15]. The phenotype and the stages observed in the knockout mice are different from those that we found here by the perturbations of the Notch pathway. No effect of the postnatal perturbation of Notch signaling on MCs suggests that there may be at least two stages, Notch-dependent early and Notch-independent later stages, in the dendritic development of MCs. At P9, many MCs did not show the mature morphology in *Maml1*^+/−^ mice, raising the possibility that maturation of MCs may be delayed in the mutants. Even at P35, however, significant number (7.9 ± 0.7%) of MCs (*n* = 38 cells from 3 mice) had a primary dendrite with tufts in two glomeruli in *Maml1*^+/−^ mice, whereas no MCs did in *Maml1*^+/+^ mice (*n* = 41 cells from 4 mice) (S6 Fig). This finding may imply that the P9 mutant phenotype was not simply caused by delayed maturation of MCs. The Notch-dependent early stage may be necessary for reducing dendritic complexity so that the mature morphology of MCs could be properly shaped at the Notch-independent later stage.

The effects of Notch on dendrites in the cerebral cortex have been complicated. Notch has been reported to have a positive effect on the length and complexity of dendrites *in vivo* [41] and a negative effect on the length of neurites, probably containing both axons and dendrites, *in vitro* [7,8]. The complexity of neurites has been affected by Notch negatively [7] and positively [8]. These discrepancies may be explained by the pleiotropic functions of Notch and numerous neuronal types, which have not been examined, and by the non-canonical Notch pathway, which has been shown to control the volume of glomeruli in *Drosophila* [39]. The distinction of neuronal types and stages in which Notch works may be necessary. It remains to be determined if the Notch-dependent early stage is unique to MCs or used in other neuronal types.

## Materials and Methods

### Animals

All experimental procedures were approved by the Animal Care and Use Committee (Chiba University) and conducted in accordance with the Guidelines for Use of Laboratory Animals (Japan Neuroscience Society). ICR and C57BL/6J mice were purchased from CLEA, Japan. Generation of *Maml1*-deficient mice has been previously described [20,42]. Mutant mice were backcrossed to C57BL/6J mice at least 10 times before use in the experiments. The noon of the day when a vaginal plug was detected was designated E0.5. The day of birth was designated P0.

### Size analysis of the brain, haematoxylin–eosin (HE) staining and DiI labeling

Dissected brains were placed horizontally, and the lengths and widths of the OB and cerebral cortex were measured along and perpendicularly to the longitudinal fissure, respectively. For HE staining, the brains were fixed in 6% formalin neutral buffer solution (pH 7.4), embedded in paraffin and sectioned sagittally at 6 μm. The sections were de-waxed, re-hydrated with decreasing concentrations of ethanol and stained with HE. For DiI labeling, the brains were fixed in 4% paraformaldehyde in phosphate-buffered saline (PBS), and a small crystal of DiI (Life Technologies) was placed superficially into the LOT. They were incubated in PBS containing 1 μM leupeptin (Roche) at 37°C, embedded in 3% low-melting-point agarose (Lonza) in PBS and coronally sectioned at 100 μm using a vibratome (Dosaka EM).

### Plasmids

pCAG-EYFP [23] and pCAG-rtTA [25] carry *EYFP* and *rtTA*, respectively, downstream of the CAG promoter. pTRE-mCherry was purchased from Clontech. pTRE-mCherry, pTRE-EYFP [25], pTRE-ECFP and pTRE-dnMAML1-EGFP carry *mCherry*, *EYFP*, *ECFP* [23] and *dnMAML1-EGFP* [26], respectively, downstream of TRE. pTRE-caNotch-Venus carries *caNotch*, which lacks the coding region of the extracellular domain of Notch1 [23], an internal ribosomal binding site and *Venus* [43] downstream of TRE. pTRE-dnMib1-EYFP carries *dnMib1*, which encodes only the N-terminal 767 amino acids but not the C-terminal RING fingers of the mouse Mib1 and acts dominant-negatively [31,44], fused with *EYFP* in frame, downstream of TRE.

### *In vivo* electroporation

To transfect DNA into a limited portion of the nervous system, we used *exo utero* electroporation (S2A Fig), in which electrodes are exactly placed over a targeted site of the clearly visible embryo in the yolk sac. E11.5 was chosen, because electroporation at this stage was the most effective for transfection into MCs and OSNs. E11.5 ICR mouse embryos were electroporated as described [23,24], with slight modifications. In brief, a pregnant mouse was anesthetized with intraperitoneal injection of the solution (20 μl per g body weight) that contained 15 μg/ml medetomidine (Kyoritsu Seiyaku), 160 μg/ml midazolam (Astellas Pharma) and 250 μg/ml butorphanol tartrate (Meiji Seika Pharma) in saline. For misexpression in MCs and OSNs, DNA was injected into the rostral portion of the telencephalon and the nasal cavity using a microinjection needle (“m” in S2A Fig and Fig 10A), respectively. Then, five square pulses of 50-ms duration with a 950-ms interval at 22 V were delivered by placing the electrodes as illustrated in the figures.

0.5 mg/ml (140 nM) pCAG-EYFP was electroporated to examine transfectability in S2 Fig and Fig 10B. To test strict control of inducible expression in S3 Fig, 0.1 mg/ml (28 nM) pCAG-EYFP as a transfection indicator, 0.25 mg/ml (68 nM) pCAG-rtTA and 0.42 mg/ml (136 nM) pTRE-mCherry were transfected.

For inducible misexpression of *caNotch* or *dnMAML1* in Figs 3, 6 and 7, 0.25 mg/ml (68 nM) pCAG-rtTA, 0.25 mg/ml (86 nM) pTRE-ECFP and 0.9 mg/ml (172 nM) pTRE-caNotch-Venus or 0.5 mg/ml (172 nM) pTRE-dnMAML1-EGFP were transfected. 0.25 mg/ml (68 nM) pCAG-rtTA and 0.5 mg/ml (172 nM) pTRE-ECFP were transfected as a control.

For inducible misexpression of *dnMib1* in Figs 8 to 10, 0.25 mg/ml (68 nM) pCAG-rtTA, 0.11 mg/ml (43 nM) pTRE-EYFP and 0.7 mg/ml (172 nM) pTRE-dnMib1-EYFP were transfected. 0.25 mg/ml (68 nM) pCAG-rtTA and 0.44 mg/ml (172 nM) pTRE-EYFP were transfected as a control.

The concentrations of pCAG-rtTA and pTRE-vectors were determined for efficient induction in the presence of Dox and for repression in the absence of Dox as described previously [25]. Dox (Clontech) was administered to pregnant mice via drinking water containing 2 mg/ml Dox. For P4 pups, Dox was intraperitoneally injected at the dose of 100 μg per 30 g of body weight [25].

### Cell culture

Dissociated neurons were prepared from the OB of ICR mouse embryos and cultured as described previously [30] with slight modifications. In brief, after trypsinization and gentle trituration of the OB, cells were plated at a density of 3.0 × 10^3^ cells/cm^2^ onto polyethylenimine-coated glass coverslips [45]. Half of the media was replaced at 1 DIV and 4 DIV. At 6 DIV, Nalm-6 human B-cell acute lymphoblastic leukemia cells overexpressing Jag1 or control cells that were X-irradiated with 600 rad [29] were added at a density of 2.5 × 10^5^ cells/cm^2^. Cells on the coverslips were fixed at 8 DIV.

### Immunofluorescence

Immunohistochemistry was performed as described for sections [11] and cells on the coverslips [46] with slight modifications. Brains were coronally sectioned at 60 μm (for dendrite analyses), 30 μm (for EYFP/Tbx21 immunostaining) or 16 μm (for EYFP/NICD1/Tbx21 triple and Tbr1 immunostaining) with a cryostat (Leica Biosystems). Sections were immunostained with antibodies: rat monoclonal anti-GFP (1:1,000, D153-3, MBL) for EYFP and ECFP, rabbit anti-Notch1 ICD (1:100, #2421, Cell Signaling Technology), mouse monoclonal anti-Tbx21 (1:100, SC-21749, Santa Cruz) and rabbit anti-Tbr1 (1:500, AB10554, Millipore). Goat polyclonal anti-MAP2 (1:200, MAP2-Go-Af860, Frontier Institute), mouse monoclonal anti-Tau1 (1:500, clone PC1C6, Millipore) and rabbit polyclonal anti-Tbx21 (1:100, SC-21003, Santa Cruz) were used for the coverslips. Secondary goat and donkey antibodies (Thermo Fischer Scientific) were used to visualize the signals for sections: dendrite analyses, Alexa Fluor 546-conjugated anti-rat IgG; EYFP/Tbx21 staining, Alexa Fluor 488-conjugated anti-rat IgG and Alexa Fluor 633-conjugated anti-mouse IgG; EYFP/NICD1/Tbx21 staining, Alexa Fluor 488-conjugated anti-rat IgG, Alexa Fluor 633-conjugated anti-rabbit IgG and Alexa Fluor 546-conjugated anti-mouse IgG; Tbr1 staining, Alexa Fluor 546-conjugated anti-rabbit IgG, and for the coverslips: Alexa Fluor 488-conjugated anti-goat IgG, Alexa Fluor 594-conjugated anti-mouse IgG and Alexa Fluor 350-conjugated anti-rabbit IgG, respectively.

### Image analysis and quantification

Large views of the brains were obtained with a fluorescence stereomicroscope (MZ16F, Leica). Images of HE-stained sections and cell cultures were obtained with a microscope (BX60, Olympus). Other fluorescent images were acquired on the LSM5 Exciter confocal microscope (Zeiss).

Because MCs are not generated synchronously [14], we focused on MCs in the middle portion along the anteroposterior axis of the dorsomedial OB, which are mainly born at earlier stages [14], although the same phenotypes were observed throughout the OB. 3D reconstruction and dendrite analyses were performed using the AutoPath and AutoDepth modes of IMARIS FilamentTracer 7.4 (Bitplane).

To quantify NICD1 signals in the MC layer, mean fluorescence intensity (FI) of Alexa Fluor 633 (reacting with anti-NICD1) in Tbx21-positive-nuclei was measured using the Histo tool in the ZEN software (Zeiss) with the confocal setting in which the pixel intensity of the brightest sample was below saturation. As internal controls, NICD1-positive Tbx21-negative cells below the MC layer that may be migrating neurons as described previously [47,48] were used. The mean background FI was obtained by measuring Alexa Fluor 633-negative areas on the same section. The relative FI of NICD1 of each Tbx21-positive cell was defined as 100 times the ratio of the background-subtracted mean Alexa Fluor 633 intensity of the cell to the average of the background-subtracted mean Alexa Fluor 633 intensities of the internal controls on the same sections. The data in Fig 10 were obtained from three brains each for misexpresion of *EYFP* (control) and *dnMib1*.

### Behavioral test

We have developed a homing behavior test by modifying unidirectional homing tests [35,36]. After mating of a *Maml1*^+/+^ C57BL/6J female with a *Maml1*^+/−^ C57BL/6J male, a pregnant female was housed individually. At P5, the dam and its litter were transferred to a clean cage containing unused wood chips. The littermates did not undergo any previous behavioral tests. At P9, the naive littermates and wood chips of their home cage were used. A pup was placed with its body axis parallel to the demarcation lines in the middle of a disposable 20.0-cm × 13.5-cm plastic cage (ICM) on a sheet of paper demarcated into three areas. The areas where plastic dishes containing 0.2 g of wood chips from the home cage and unused chips (as a control) were placed were designated H and C, respectively. The M area was between H and C. The cumulative time spent by the pup’s snout in each area was measured in a 3 min test. Each pup was tested only once. The plastic cage was replaced with a new one after every test. The ANY-maze video tracking system (Stoelting) was also used to analyze the pup’s movement as shown in Fig 11B. Walking was evaluated by measuring the time taken to walk 5 cm. After the test, the genotypes of pups were determined by PCR.

### Statistics

Data in the text are mean ± SD. P-values were determined using Mann–Whitney U test.

## Supporting Information

S1 Fig No overt abnormalities in the OB of *Maml1* mutants at E18.5. (**A**) Whole-mount dorsal views of the OB and cerebral cortex (Cx). (**B, C**) The lengths and widths of the OB (**B**) and Cx (**C**) were indistinguishable among *Maml1*^+/+^ (*n* = 4), *Maml1*^+/−^ (*n* = 4) and *Maml1*^-/-^ (*n* = 5). (**D**) HE staining of sagittal sections of the OB was also indistinguishable among *Maml1*^+/+^, *Maml1*^+/−^ and *Maml1*^-/-^ (*n* = 3 mice of each genotype). Arrows indicate the MC layer. Scale bars: 1 mm in **A**; 0.5 mm in **D**.
(TIF)

S2 Fig Efficient transfection into MCs by *exo utero* electroporation at E11.5. (**A**) Schematic illustration of transfection into MCs. Microinjection needle (m) and electrodes are depicted. (**B, C**) Ventral view of the brain (**B**) and a coronal section of the OB immunostained for EYFP and Tbx21 (**C**), 7 days after electroporation of *EYFP* at E11.5. An arrow indicates the LOT positive for EYFP. Similar immunostaining patterns were reproducibly observed in all examined embryos (*n* = 3 mice). The OB and LOT were strongly positive for EYFP. Most EYFP-positive cells were aligned in the MC layer and were positive for Tbx21, as previously described by Imamura and Greer [47], who performed *in utero* electroporation. Scale bars: 0.5 mm in **B**; 10 μm in **C**.
(TIF)

S3 Fig Dox-specific gene induction in MCs. (**A**) Illustration of EP of CAG-*rtTA*, TRE-*mCherry* and CAG-*EYFP* (as a transfection indicator) at E11.5, and induction by Dox at E16.5. (**B**) Coronal sections of the dorsomedial OB at E18.5, after Dox administration in drinking water without (control) or with Dox (+Dox). No mCherry expression was detected in control embryos (*n* = 5). Dox-specific *mCherry* induction was reproduced in all embryos treated with Dox (*n* = 5). Scale bar: 20 μm.
(TIF)

S4 Fig No gross abnormalities in the *Maml1*^+/−^ OB at P9. (**A**) Whole-mount dorsal views of the OB and Cx. (**B, C**) The lengths and widths of the OB (**B**) and Cx (**C**) were indistinguishable between *Maml1*^+/+^ (*n* = 12) and *Maml1*^+/−^ (*n* = 14). (**D**) HE staining of sagittal sections of the OB was also indistinguishable between *Maml1*^+/+^ and *Maml1*^+/−^ (*n* = 3 mice of each genotype). Arrows indicate the MC layer. Scale bars: 1 mm.
(TIF)

S5 Fig NICD1 signals in MCs during development. Coronal sections of the OB at E15.5 (**A**), E18.5 (**B**) and P4 (**C**), immunostained for Tbr1 (**A**), Tbx21 (**B, C**) and NICD1 (**A, B, C**). MCs and their postmitotic precursors have been shown to be positive for Tbr1 [47]. NICD1-positive cells, which may be migrating neurons as described previously [47,48], were observed below the MC layer at E15.5 and E18.5 (arrows). The ventricle is to the bottom. Strong NICD1 signals were observed in the cerebral cortex of the same P4 brain on the same slide glass (the bottom panel in **C**). Similar immunostaining patterns were reproducibly observed in all examined embryos (*n* = 3 mice). Scale bar: 20 μm.
(TIF)

S6 Fig Abnormal dendrites of MCs in *Maml1*^+/−^ mice at P35. Representative images of MCs that had a primary dendrite with tufts in single (left panel) and double (right panel) glomeruli in *Maml1*^+/−^ mice at P35. Scale bar: 50 μm.
(TIF)

S7 Fig MC dendrites extending into two glomeruli in the *Maml1*^+/−^ OB at P9. A representative image of dendrites extending into two glomeruli (arrows) that branched in the deep external plexiform layer. Scale bar: 50 μm.
(TIF)
