# Supplementary figures and images for: Olfactory Sensory Neurons Control Dendritic Complexity of Mitral Cells via Notch Signaling
Source: PLoS Genet. 2016 Dec 27;12(12):e1006514. doi: 10.1371/journal.pgen.1006514 (PMC5189955; doi:10.1371/journal.pgen.1006514)

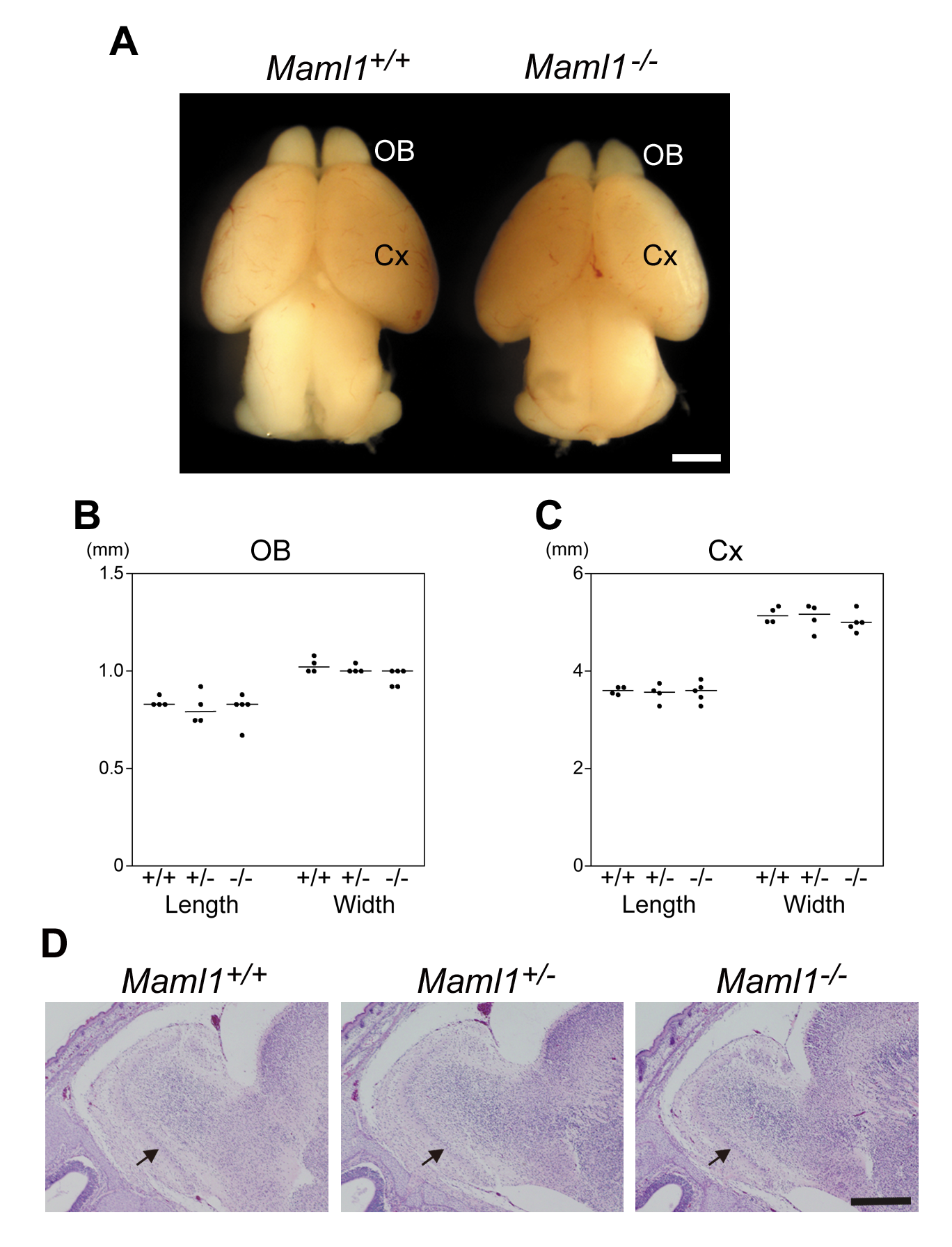

Supplement: S1 Fig — (A) Whole-mount dorsal views of the OB and cerebral cortex (Cx). (B, C) The lengths and widths of the OB (B) and Cx (C) were indistinguishable among Maml1+/+ (n = 4), Maml1+/− (n = 4) and Maml1-/- (n = 5). (D) HE staining of sagittal sections of the OB was also indistinguishable among Maml1+/+, Maml1+/− and Maml1-/- (n = 3 mice of each genotype). Arrows indicate the MC layer. Scale bars: 1 mm in A; 0.5 mm in D. (TIF) [file pgen.1006514.s001.tif]

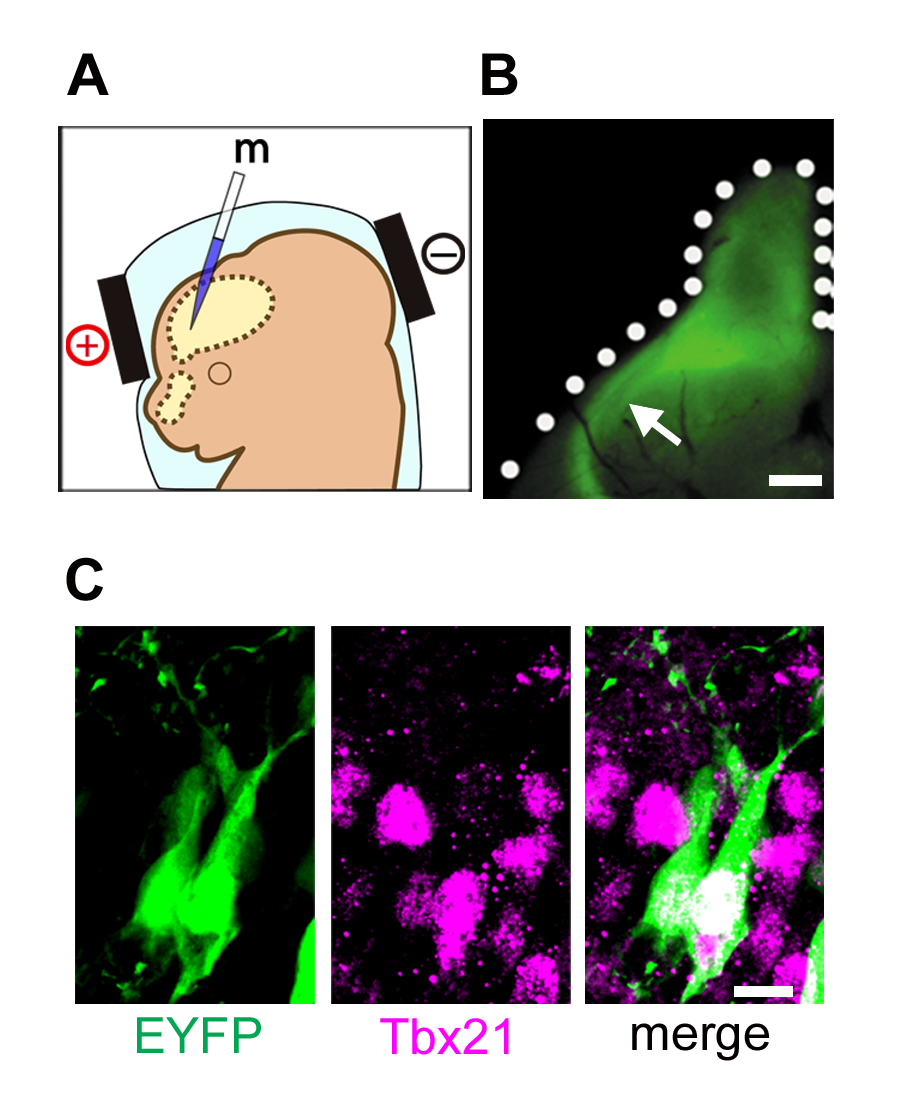

Supplement: S2 Fig — (A) Schematic illustration of transfection into MCs. Microinjection needle (m) and electrodes are depicted. (B, C) Ventral view of the brain (B) and a coronal section of the OB immunostained for EYFP and Tbx21 (C), 7 days after electroporation of EYFP at E11.5. An arrow indicates the LOT positive for EYFP. Similar immunostaining patterns were reproducibly observed in all examined embryos (n = 3 mice). The OB and LOT were strongly positive for EYFP. Most EYFP-positive cells were aligned in the MC layer and were positive for Tbx21, as previously described by Imamura and Greer [47], who performed in utero electroporation. Scale bars: 0.5 mm in B; 10 μm in C. (TIF) [file pgen.1006514.s002.tif]

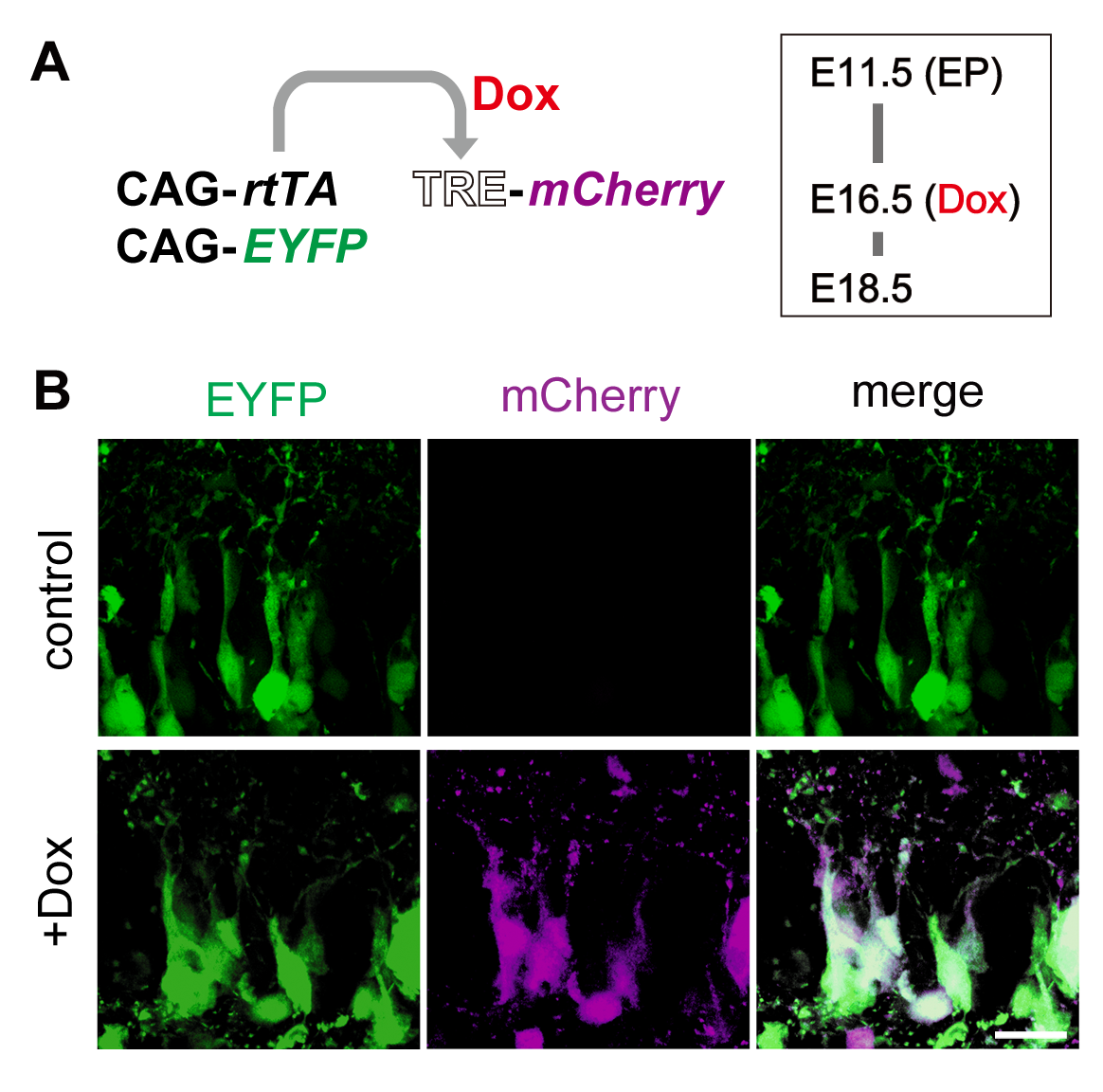

Supplement: S3 Fig — (A) Illustration of EP of CAG-rtTA, TRE-mCherry and CAG-EYFP (as a transfection indicator) at E11.5, and induction by Dox at E16.5. (B) Coronal sections of the dorsomedial OB at E18.5, after Dox administration in drinking water without (control) or with Dox (+Dox). No mCherry expression was detected in control embryos (n = 5). Dox-specific mCherry induction was reproduced in all embryos treated with Dox (n = 5). Scale bar: 20 μm. (TIF) [file pgen.1006514.s003.tif]

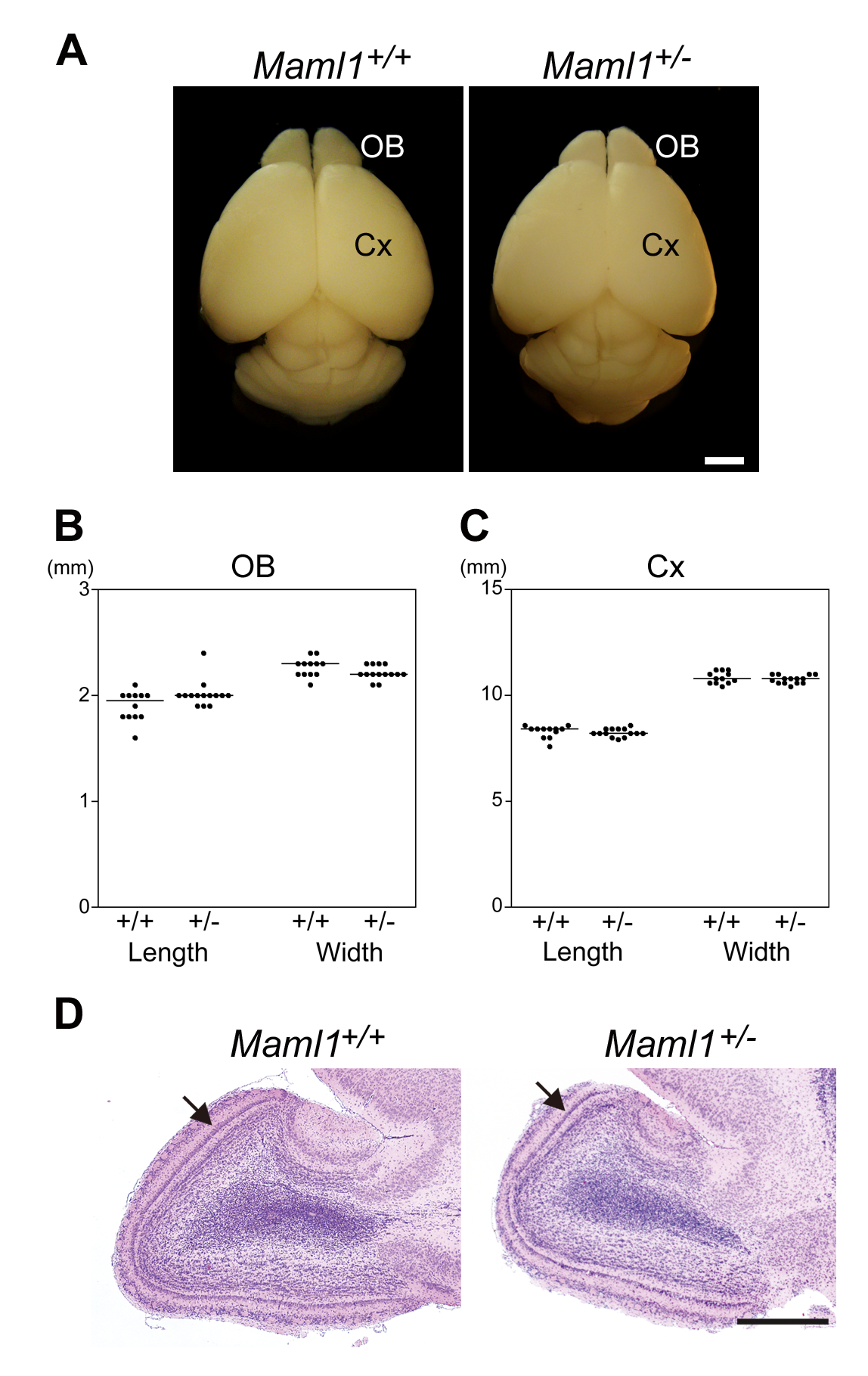

Supplement: S4 Fig — (A) Whole-mount dorsal views of the OB and Cx. (B, C) The lengths and widths of the OB (B) and Cx (C) were indistinguishable between Maml1+/+ (n = 12) and Maml1+/− (n = 14). (D) HE staining of sagittal sections of the OB was also indistinguishable between Maml1+/+ and Maml1+/− (n = 3 mice of each genotype). Arrows indicate the MC layer. Scale bars: 1 mm. (TIF) [file pgen.1006514.s004.tif]

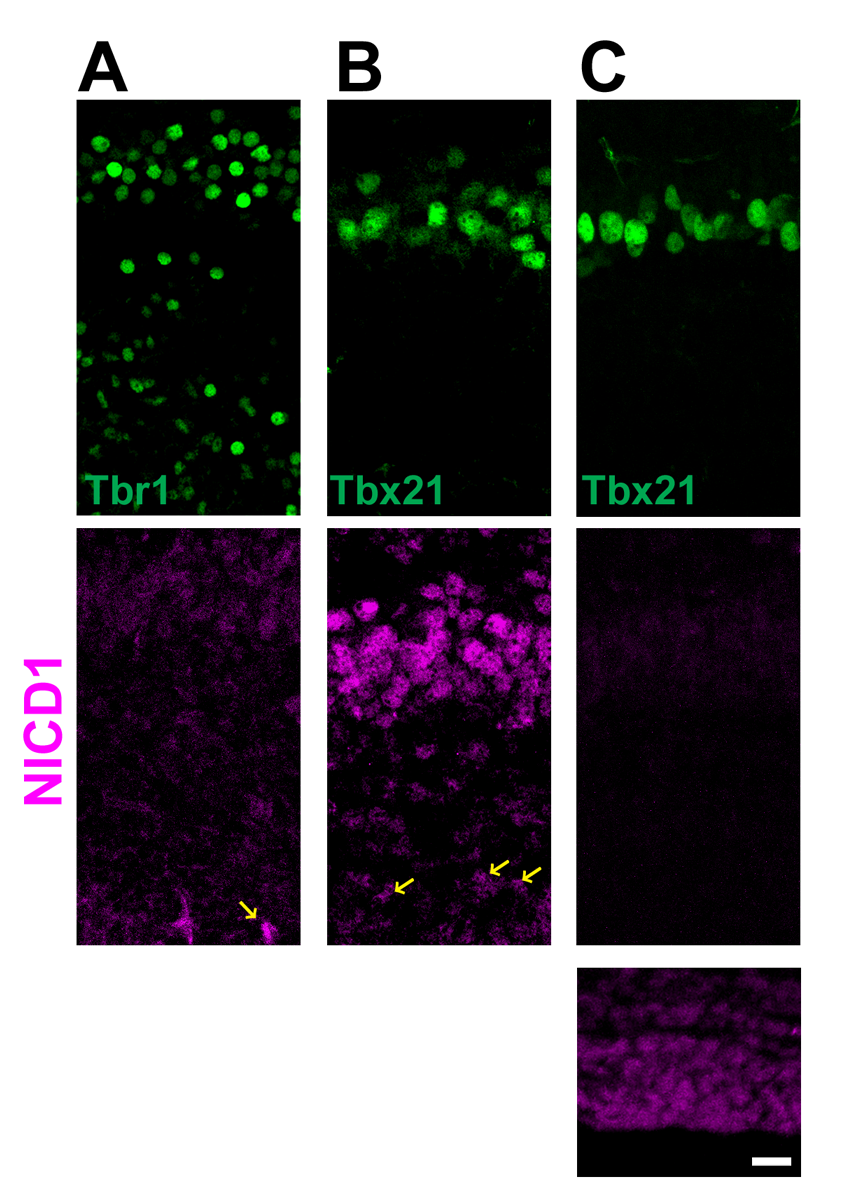

Supplement: S5 Fig — Coronal sections of the OB at E15.5 (A), E18.5 (B) and P4 (C), immunostained for Tbr1 (A), Tbx21 (B, C) and NICD1 (A, B, C). MCs and their postmitotic precursors have been shown to be positive for Tbr1 [47]. NICD1-positive cells, which may be migrating neurons as described previously [47,48], were observed below the MC layer at E15.5 and E18.5 (arrows). The ventricle is to the bottom. Strong NICD1 signals were observed in the cerebral cortex of the same P4 brain on the same slide glass (the bottom panel in C). Similar immunostaining patterns were reproducibly observed in all examined embryos (n = 3 mice). Scale bar: 20 μm. (TIF) [file pgen.1006514.s005.tif]

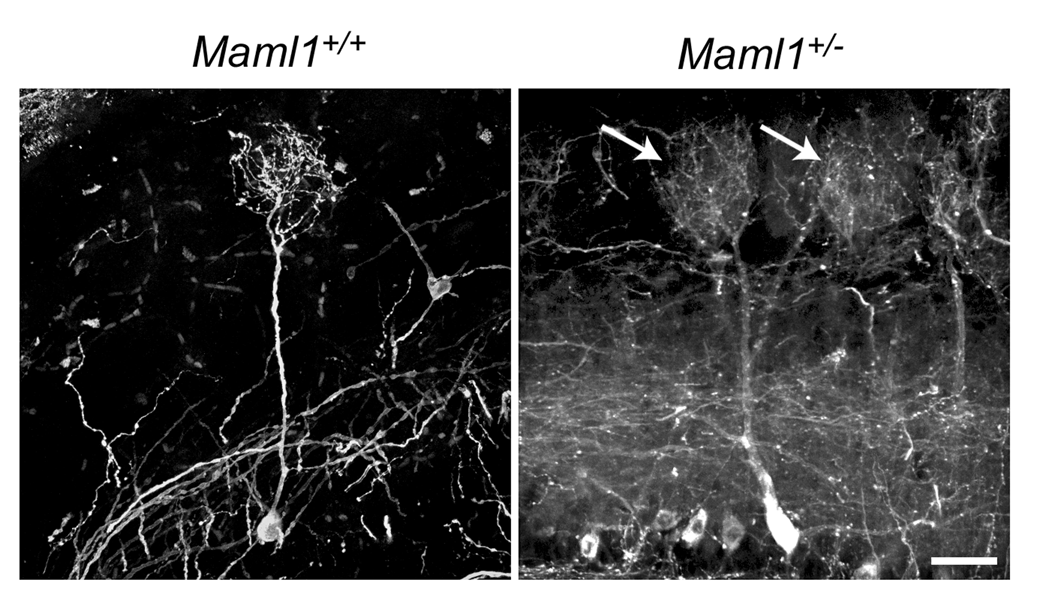

Supplement: S6 Fig — Representative images of MCs that had a primary dendrite with tufts in single (left panel) and double (right panel) glomeruli in Maml1+/− mice at P35. Scale bar: 50 μm. (TIF) [file pgen.1006514.s006.tif]

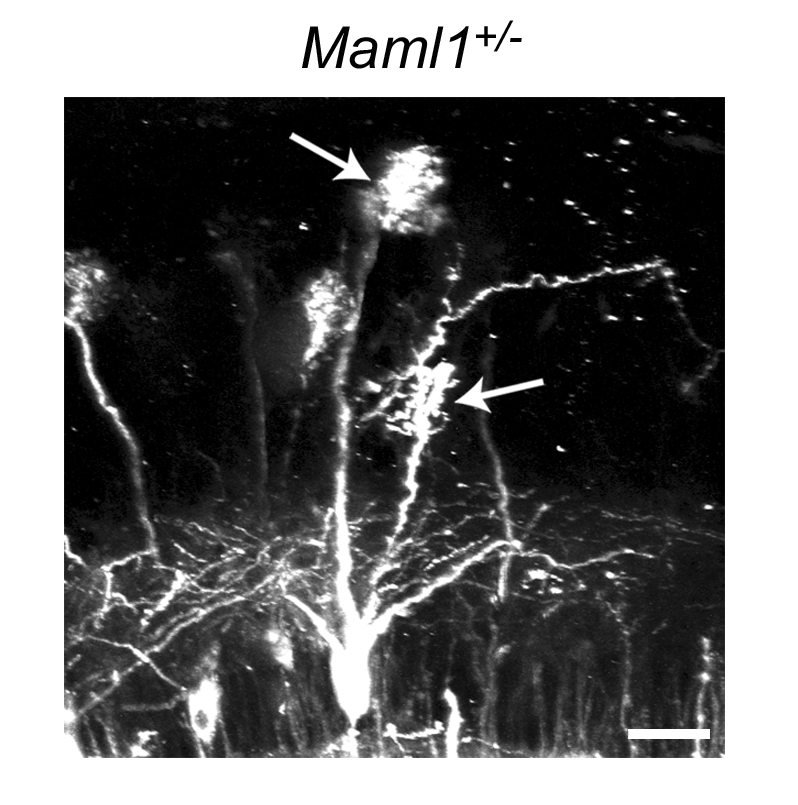

Supplement: S7 Fig — A representative image of dendrites extending into two glomeruli (arrows) that branched in the deep external plexiform layer. Scale bar: 50 μm. (TIF) [file pgen.1006514.s007.tif]
